# Postbiotic effects of *Enterococcus faecium* JB00008 on gut health and IBD vaccination in broiler chickens

**DOI:** 10.1371/journal.pone.0353974

**Published:** 2026-07-16

**Authors:** Seonju Lee, Seojin Choi, Jongbin Park, Biao Xuan, Eun Bae Kim

**Affiliations:** 1 Department of Applied Animal Science, College of Animal Life Sciences, Kangwon National University, Chuncheon, Republic of Korea; 2 Institute of Animal Life Science, Kangwon National University, Chuncheon, Republic of Korea; Tokat Gaziosmanpaşa University: Tokat Gaziosmanpasa Universitesi, TÜRKIYE

## Abstract

Feeding various probiotic lactic acid bacteria, including *Enterococcus faecium*, can alleviate intestinal inflammation and improve gut health in animals. Recently, postbiotics—non-living preparations derived from microbial cells or their metabolites—have gained attention. However, studies on the effects of these postbiotics on immune markers and changes in the gut microbiota of chickens are limited. In this study, we evaluated the effects of the probiotic strain *E. faecium* JB00008 on the chicken intestinal tract and characterized immune markers and gut microbiota following viral vaccination. Chicks were divided into three groups (Control, DH5α, and JB00008) and administered the respective supernatants in drinking water from days 1–12 at a 3:7 ratio. Samples were collected on days 13 and 28 for microbiota and gene expression analyses. To immunize against infectious bursal disease (IBD), the chicks received an oral vaccine on day 13. Growth, immune, and gut parameters were measured. Body weights did not differ among groups (*p* = 0.380). Several intestinal immune markers—mucin 2 (*MUC2*, *p* = 0.001), occludin (*OCLN*, *p* < 0.001), and interleukin-10 (*IL-10*, *p* < 0.001)—were significantly higher in the JB00008 group. Annexin A5 (*ANXA5*, *p* = 0.005) and interleukin-6 (*IL-6*, *p* < 0.001) also differed among groups. After IBD vaccination, IBD-specific immunoglobulin A (IgA, *p* = 0.200) and IgG (*p* = 0.065) responses were comparable; however, the alpha (*p* < 0.001) and beta diversities (*p* = 0.001) were significantly different among the groups. The JB00008 group showed higher *Enterococcus* and *Bifidobacterium*, with enrichment of pathways associated with iron complex transport systems (*p* < 0.050). These findings suggest that JB00008 postbiotics may enhance intestinal barrier function and microbiota health without affecting growth, thereby supporting gut stability after vaccination. Furthermore, these results highlight the potential use of *E. faecium* JB00008 as a feed additive and vaccine adjuvant.

## Introduction

Probiotics are live microorganisms that, when administered in adequate amounts, confer health benefits to the host. With growing consumer demand for antibiotic-free poultry products, the use of probiotics in poultry farming has increased significantly [1]. Among them, *Enterococcus faecium* supplementation in broiler diets reduces daily feed intake without affecting body weight or average daily gain [2]. Additionally, certain probiotic strains have demonstrated the ability to alleviate intestinal inflammation and prevent epithelial cell apoptosis, thereby contributing to the maintenance of intestinal barrier integrity [3]. However, these effects are strain-specific, necessitating an evaluation of both the strains and their resulting postbiotics. This transition is increasingly important, as postbiotics—the functional metabolites of probiotics—offer a more stable and safer alternative for enhancing poultry immunity [4].

The International Scientific Association of Probiotics and Prebiotics (ISAPP) defined postbiotics as “non-living preparations” that are “relating to or resulting from living organisms [5].” In other words, postbiotics refer to bioactive compounds generated after microbial cells have been inactivated or died. These include structural fragments such as cell wall components, metabolic byproducts, and proteins [6]. Postbiotics can lower the intestinal pH, thereby suppressing the growth of pathogenic bacteria and promoting the proliferation of beneficial microorganisms such as *Lactobacillus* and *Bifidobacteria* [7]. With the growing interest in antibiotic alternatives, postbiotics are increasingly being investigated as functional feed additives aimed at improving the health and growth performance of monogastric animals, such as poultry and swine.

Meanwhile, the administration of postbiotics, such as cell-free supernatants, has been reported to improve intestinal barrier function and enhance growth performance in poultry [8]. Among these, postbiotics derived from *Enterococcus faecium* have shown beneficial effects, including enhanced growth performance, improved digestive efficiency, strengthened immunity, and reduced incidence of diarrhea [9]. These findings highlight the need to further explore the influence of *E. faecium* postbiotics on the gut microbiota composition and host immune responses [10].

Therefore, there is an increasing need for postbiotics to enhance the intestinal health and prevent mucosal infections in chickens. In this study, we investigated the effects of *Enterococcus faecium* JB00008, which was previously studied as a probiotic, when applied as a postbiotic in chickens. Specifically, we evaluated the effects of JB00008 postbiotics on the expression of intestinal genes, including IL-10 [11], a cytokine involved in the maintenance and restoration of the intestinal epithelium, and MUC2 [12], a major component of the mucus layer that protects the small and large intestines, as well as on the composition of the gut microbiota. Therefore, this study aimed to evaluate the potential of JB00008 postbiotics as a functional feed additive to support intestinal health and explore their potential association with host immune modulation in poultry.

## Materials and methods

### Animal trial

A total of 60 male Ross 308 broiler chicks were purchased from the Samhwa Breeding Farm (Hongseong, Korea) and raised at the Kangwon National University Experimental Poultry Facility at a stocking density of 12.3 birds/m^2^. The chicks were housed in cages with 10 birds per cage, with 2 replicate cages per treatment group (20 birds per treatment). Environmental conditions, including temperature and humidity, were continuously monitored from days 1–14 using a microcontroller-based system (NodeMCU). The average temperature during this period was 28.04 ± 1.52 °C (range: 25.80–30.16 °C), and the average relative humidity was 50.09 ± 9.17% (range: 42.93–77.40%). The chicks were provided ad libitum access to a commercial starter diet until day 14, after which they were switched to a pelleted grower diet (S1 Table). All experimental procedures involving animals were conducted in accordance with the guidelines approved by the Institutional Animal Care and Use Committee (IACUC) of Kangwon National University (Approval No. KW-220905–1).

To minimize potential pain and distress during experimental procedures, all treatments were administered via oral gavage, a non-invasive method associated with minimal pain. Consequently, no anesthesia or analgesia was utilized during the administration. This procedure causes negligible pain when performed by trained personnel, and the use of such agents was deemed unnecessary; moreover, anesthesia could introduce additional physiological stress or confounding effects on the experimental outcomes. All procedures were conducted by highly experienced researchers who handled the birds with extreme care to reduce handling stress and avoid any physical injury.

Humane endpoints were predefined to minimize potential suffering. Birds were monitored at least once daily for general health status, behavior, posture, and access to food and water. The criteria for humane endpoints included severe body weight loss (more than 20% compared to the group average), severe lethargy, or the appearance of abnormal clinical lesions. If any bird had reached these criteria, it would have been euthanized immediately via CO_2_ inhalation to prevent further distress. In this study, no birds exhibited such clinical signs, and no unexpected mortality occurred. Scheduled euthanasia was performed on days 13 and 28 using CO_2_ inhalation followed by cervical dislocation as a confirmatory method, and animal carcasses were disposed of as medical waste in accordance with institutional regulations.

### Oral administration of postbiotics and oral vaccine

*Escherichia coli* DH5α and *Enterococcus faecium* JB00008, a strain isolated from cheonggukjang with notable antibacterial activity [10, 13], were selected as sources for postbiotic production. While *E. faecium*, previously characterized as a probiotic strain, was employed to evaluate its functional benefits in postbiotic form, *E. coli* DH5α was included as a representative Gram-negative bacterium to serve as a comparative reference. This selection allowed us to examine whether the effects of postbiotics on the intestinal environment are strain- or taxa-dependent. In addition, based on our previous observations that supernatants derived from lactic acid bacteria significantly upregulate M cell markers in chickens [14], E. coli-derived supernatant was included to determine whether similar immunomodulatory effects are conserved across phylogenetically distinct bacterial groups. The strains were cultured in Luria-Bertani (LB) broth and de Man, Rogosa and Sharpe (MRS) broth, at 37 °C for 10 h, respectively. Following incubation, the cultures were centrifuged at 3,134 × g for 10 min and only the supernatant was collected. The supernatants were filtered through a 0.2 µm syringe filter (Minisart®, Sartorius) to ensure sterility. Each filtered supernatant (150 mL) was then mixed with 350 mL of sterilized tap water and provided as drinking water to the broilers from day 1 to day 12 between 9:00 a.m. and 7:00 p.m. Outside this time window, all groups were provided with fresh water only, which was replenished daily and offered ad libitum.

**Fig 1:**
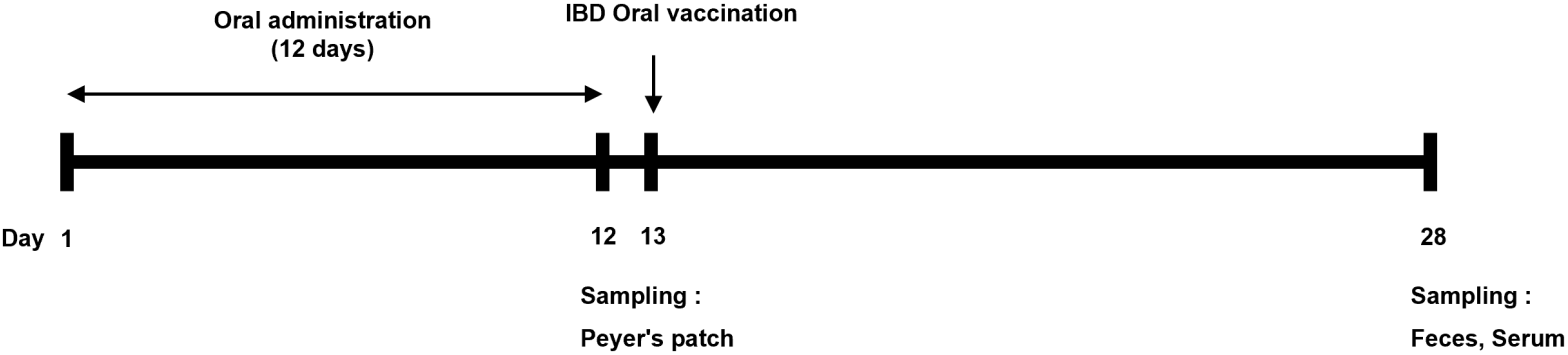
Time Schedule for the Chicken IBD Vaccine Experiment. Postbiotics were administered from Days 1 to 12. Oral administration of the infectious bursal disease (IBD) vaccine began at 13 days of age. Peyer's patches were collected on Day 13, and feces and serum were collected at Day 28.

On day 13, a commercial infectious bursal disease (IBD) vaccine (PoulShot IB-Castle, ChoongAng Vaccine Laboratory, Korea) was administered orally in accordance with the manufacturer’s instructions. Briefly, feed and water were withdrawn 2 h prior to vaccination (Fig 1). The vaccine was dissolved in room-temperature drinking water (20–24 °C) and administered orally.

### Sample collection

On day 13, Peyer’s patches were carefully dissected from the ileal mucosa of broiler chickens using sterile dissection forceps. Tissue segments (1–2 cm proximal to the ileocecal valve, standardized to ~1 cm in length) were collected to ensure anatomical consistency, and used for subsequent qPCR analysis. All dissection tools and surfaces were sterilized with 70% ethanol between uses. To preserve molecular integrity, samples were immediately frozen in liquid nitrogen and transported to the laboratory. They were subsequently stored at −80°C until RNA extraction.

Total RNA was extracted from the ileal tissues using TRIzol® reagent (Thermo Fisher Scientific, USA) according to the manufacturer’s instructions. On day 28, a key economic indicator in the broiler industry [15], individual body weights were measured using a digital scale (±0.1 g accuracy). On day 13, all 10 birds from the first of the two replicate cages within each treatment group were sampled for molecular and immunological analyses. The remaining second cage was maintained until day 28 for subsequent intestinal microbiota analysis, thereby accounting for the sampling loss. Body weights were recorded exclusively on the designated sampling days rather than weekly, and growth parameters were calculated based on the actual number of birds remaining in each period to account for the sampling loss. Prior to fecal collection, birds were weighed and moved to sanitized plastic pens lined with sterile aluminum foil. Fresh dropping were immediately sampled into 1.7 mL tubes using sterile pipette tips, and blood was subsequently collected from the wing vein using 1 mL sterile syringes. Birds were euthanized using the same CO_2_ protocol as that used on day 13. All collected samples, including feces and serum, were immediately snap-frozen in liquid nitrogen and stored at −80 °C until further analysis.

### cDNA synthesis and qPCR

To evaluate the effects of postbiotic supplementation on intestinal immunity and epithelial integrity, quantitative real-time PCR (qPCR) was conducted to assess the expression of IL-10 (an anti-inflammatory cytokine), MUC2 (a protective mucin produced by intestinal goblet cells), and OCLN (a key tight junction protein). Total RNA extracted from ileal tissues was reverse transcribed into cDNA using the PrimeScript™ RT Reagent Kit (Takara Bio, Japan), according to the manufacturer’s instructions. qPCR was performed using TB Green® Premix Ex Taq™ (Tli RNaseH Plus, Takara Bio, Japan). Gene expression was analyzed for five target genes: *IL-10* (a marker of immune modulation), occludin (*OCLN*) and *MUC2* (related to tight junction integrity and mucus barrier), *ANXA5*, and *IL-6*. Target genes were selected to evaluate gut barrier integrity and immune responses, including MUC2 and OCLN as markers of mucosal protection and tight junction function, and IL-6 and IL-10 to assess pro- and anti-inflammatory responses. Notably, ANXA5 was selected as a potential M cell-related marker due to the absence of the mammalian GP2 gene in the avian genome, supported by amino acid sequence alignment identifying murine and avian ANXA5 as orthologues. Glyceraldehyde 3-phosphate dehydrogenase (GAPDH) was used as the sole housekeeping gene for normalization, and the qPCR was performed in a single run with each sample analyzed in duplicate. The primer sequences [16, 17] used for each gene are listed in Table 1. Relative mRNA expression levels were quantified using the 2^ − ΔΔCt method, and the Ct values of *IL-10, OCLN, MUC2, ANXA5*, and *IL-6* were normalized to that of *GAPDH*.

**Table 1:** Primers for small intestinal immune marker genes.

Target gene	Accession No.	Forward primer(5’-3’)Reverse primer(3’-5’)	Amplicon length(bp)	Annealing Tm(℃)
*GAPDH*	NM_204305.2	F: GTGGTGCTAAGCGTGTTATCATC	269	59.3
		R: GGCAGCACCTCTGCCATC		64.5
*IL-10*	NM_001004414.2	F: GCTGCGCTTCTACACAGATGAG	73	62.1
		R: GCCCATGCTCTGCTGATGA		58.8
*MUC2*	XM_001234581.3	F: CTGATTGTCACTCACGCCTTAATC	147	61.0
		R: GCCGGCCACCTGCAT		52.0
*OCLN*	NM_205128.1	F: CCCAGAAGACGCGCAGTAAG	61	61.4
		R: GCGCGGTCCCAGTAGATG		60.5
*ANXA5*	NM_001031538.2	F: AGTATACAAGAGGCACCGTG	252	58.2
		R: GTCTCATCAAAGATACCATC		51.8
*IL-6*	XM_015281283.1	F: CTCCTCGCCAATCTGAAGTC	100	59.4
		R: CCCTCACGGTCTTCTCCATA		59.4

Target genes, GenBank accession numbers, forward (F) and reverse (R) primer sequences (5′–3′), expected amplicon lengths, and annealing temperatures used for quantitative qPCR are shown. bp, base pairs; Tm, melting temperature.

### IgA & IgG ELISA

ELISA was performed to measure serum IgG and fecal IgA levels against infectious bursal disease virus (IBDV) using a commercial kit (BioChek, CK113) according to the manufacturer’s instructions with minor modifications. Briefly, serum samples were diluted 1:500 and fecal supernatants were diluted 1:100, then added to antigen-coated plates. After incubation at room temperature for 30 min, the plates were washed and incubated with secondary antibodies. For serum IgG detection, the HRP-conjugated secondary antibody provided in the kit was used. For fecal IgA detection, a goat anti-chicken IgA HRP-conjugated antibody (LSBio, Cat# LS-C56695) was used at a dilution of 1:10,000.

Following incubation and washing, substrate solution was added to develop color, and the reaction was stopped according to the manufacturer’s instructions. Absorbance was measured at 405 nm using a microplate reader.

### DNA extraction and sequencing for gut microbiome

Following the 28-day rearing period, DNA was extracted from 250 mg of the chicken fecal sample according to the manufacturer's protocol for the NucleoSpin Soil Kit. After DNA extraction, the DNA was stored at −20 °C. PCR of the V4 region of 16S ribosomal RNA was performed using Universal primers (forward: 5′-GGA CTACHVG GGTWTCTAAT-3′ and reverse: 5′-GTGCCAGCMGCCGCGGTA A-3′) and TaKaRa Ex-Taq polymerase (TaKaRa Bio, Shiga, Japan). PCR conditions were 3 min at 94 °C, 30 cycles of [94 °C for 45 s, 55 °C for 1 min, 72 °C for 1.5 min], followed by 10 min at 72 °C. PCR amplicon purification was performed using a QIAquick PCR Purification Kit. Each sample was then normalized to a 50 ng level using a Spark 10M Multimode microplate reader. DNA libraries were constructed and sequenced using the Illumina MiSeq platform, generating 2x300 bp paired-end reads.

### Microbiome analysis and metagenomic prediction

Microbial community analysis was conducted using Quantitative Insights into Microbial Ecology 2 (QIIME 2) v.2023.11 (https://qiime2.org) [18] in combination with the SILVA (v138) 16S rRNA gene reference database [19]. Raw sequencing reads were subjected to primer and adapter removal using the Cutadapt and demux plugins in QIIME 2, followed by demultiplexing with custom Perl scripts. Subsequent quality filtering, trimming, and removal of the chimeric sequences were performed using the denoise-paired function of the Divisive Amplicon Denoising Algorithm (DADA2) [20] plugin. For DADA2 processing, sequences were trimmed by 12 and 8 bases for forward and reverse reads respectively and truncated at 200 bp and 180 bp. Chimera removal was conducted using the consensus method with default parameters unless otherwise specified. A phylogenetic tree was constructed using the q2-phylogeny plugin to facilitate microbial diversity analysis. Alpha diversity was assessed using observed features and Faith’s phylogenetic diversity, and beta diversity was evaluated using weighted and unweighted UniFrac distances based on principal coordinate analysis (PCoA) following rarefaction to 17,000 reads.

### Prediction of metagenomic pathways

Based on 16S rRNA gene sequencing data, functional pathway predictions were generated using the q2-picrust2 plugin implemented in QIIME 2 (https://github.com/gavinmdouglas/q2-picrust2,), which is based on PICRUSt2. Amplicon sequence variants (ASVs) were placed into a reference phylogenetic tree using reference phylogenetic tree placement, followed by hidden-state prediction to infer gene family abundances. Predicted gene families were categorized into KEGG Orthology (KO) groups and subsequently mapped to metabolic pathways using the KEGG database. Pathway abundances were normalized to relative abundances prior to downstream analysis. Only pathways with sufficient prevalence across samples were retained for further statistical analysis.

### Statistical analysis

Statistical analyses were performed using the R software (version 4.4.0). To compare the mean values among the three groups, the Kruskal–Wallis test and Dunn’s test were applied, while the Mann–Whitney U test was used for pairwise comparisons between two groups. For predicted functional pathways, significant differences were identified using LEfSe analysis with an LDA score threshold of > 2.7 and p < 0.050.

Postbiotic treatments were administered at the cage level through shared drinking water, establishing the cage as the experimental unit for treatment allocation. For molecular, immunological, and microbiological analyses, sampled birds were treated as nested observational units; thus, the sample size (n) reported in the figures and tables refers to the number of individual birds analyzed. Given this nested structure and limited cage replication, these individual-level non-parametric analyses were primarily utilized for exploratory evaluation of physiological, immunological, and microbiological responses. Accordingly, the findings should be interpreted with appropriate caution and not as definitive pen-level treatment effects. Statistical significance was set at *p* < 0.050.

## Results

### Body weight and tissue gene expression

**Fig 2:**
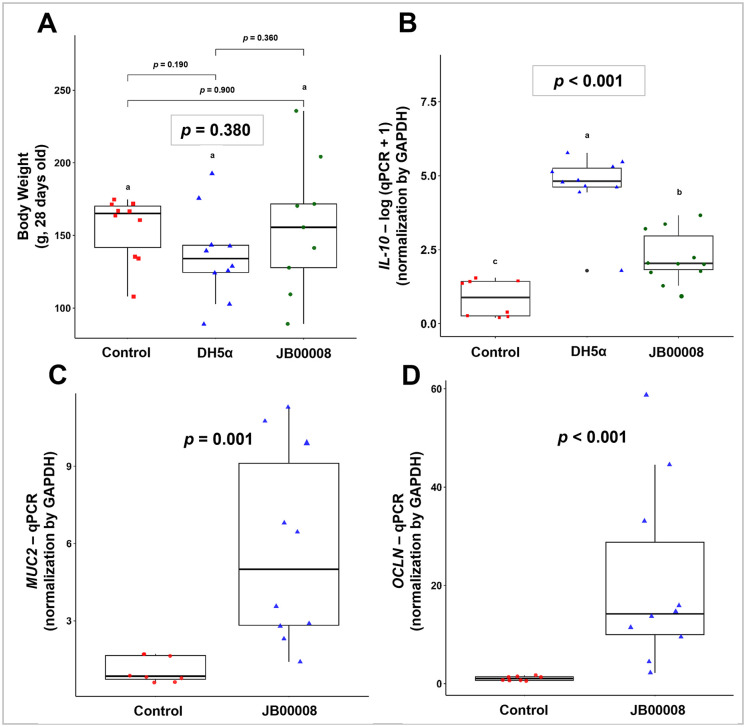
Growth and Intestinal Barrier Parameters of 28-Day-Old Chickens. (A) Body weight of 28-day-old chickens (n = 10 individual birds sampled per treatment). (B) mRNA expression of *IL-10* in Peyer’s patches of 13-day-old chickens after 12 days of oral administration of culture supernatant (Control, n = 8; DH5α, n = 10; JB00008, n = 10). (C, D) mRNA expression of *MUC2* and *OCLN* to evaluate intestinal tight junction integrity. Bars represent mean ± standard deviation. p-values were determined by Kruskal–Wallis test followed by Dunn’s post hoc test (A, B). The sample size (*n*) indicates the number of individual birds analyzed as observational units (subsamples) from the designated cage to account for individual physiological variation.

The differences in body weight, as a growth parameter, among the groups fed with PBS (Control: 155.32 ± 20.86 g), *E. coli* DH5α (136.39 ± 30.78 g), and *E. faecium* JB00008 (156.16 ± 43.19 g) were not statistically significant (*p* = 0.380). No significant differences in body weight were observed between the Control, DH5α, and JB00008 when compared pairwise. As shown in Fig 2B, *IL-10* expression was significantly elevated in both postbiotics-fed groups compared to the PBS control (*p* < 0.001), with the DH5α group exhibiting the highest expression level. Furthermore, as illustrated in Fig 2C and 2D, the expression levels of *MUC2* (*p* = 0.001) and *OCLN* (*p* < 0.001) were significantly higher in the postbiotics-treated groups than in the control. These findings indicate that although *E. faecium* JB00008 postbiotic administration did not significantly affect body weight, it positively influenced the expression of intestinal immune-related genes, suggesting a role in enhancing mucosal immunity and maintaining epithelial barrier function.

### Immunization with IBD vaccine

**Fig 3:**
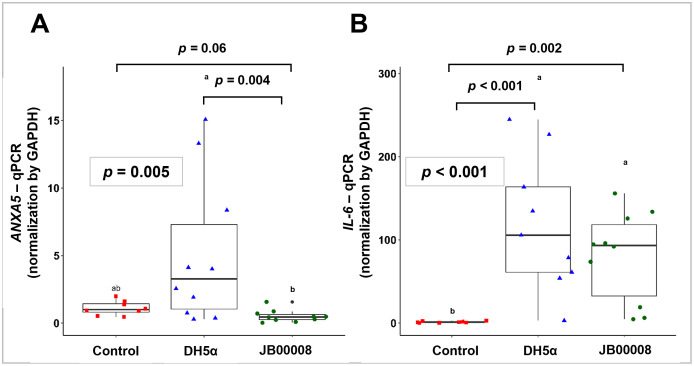
IL-6 and ANXA5 mRNA expression in peyer’s patch. mRNA expression of *ANXA5* (A) and *IL-6* (B) in Peyer’s patches of 13-day-old chickens after 12 days of oral administration of culture supernatant. Each point represents an individual bird sampled from the designated cage (Control, n = 8; DH5α, n = 10; JB00008, n = 10). p-values were determined by Kruskal–Wallis test followed by Dunn’s post hoc test. The sample size (*n*) reflects the number of individual birds analyzed as observational units (subsamples) per treatment to capture within-unit biological variation.

To evaluate the effect of *E. faecium* JB00008 postbiotics on intestinal M cells in ROSS 308 chickens, qPCR was performed using Peyer’s patch samples to detect *ANXA5* and *IL-6*. Significant differences were observed among the three groups for both *ANXA5* (*p* = 0.005) and *IL-6* (*p* < 0.001) levels. As shown in Fig 3A, ANXA5 expression did not differ significantly between the control and JB00008 groups (*p =* 0.060). However, the DH5α group showed significantly higher ANXA5 expression compared to both the control group (*p =* 0.005) and the JB00008 group (*p =* 0.004). *IL-6* expression was significantly lower in the control group compared to both JB00008 and DH5α groups (*p* < 0.001 for both), whereas no significant difference was observed between the JB00008 and DH5α groups (*p* = 0.500).

**Fig 4:**
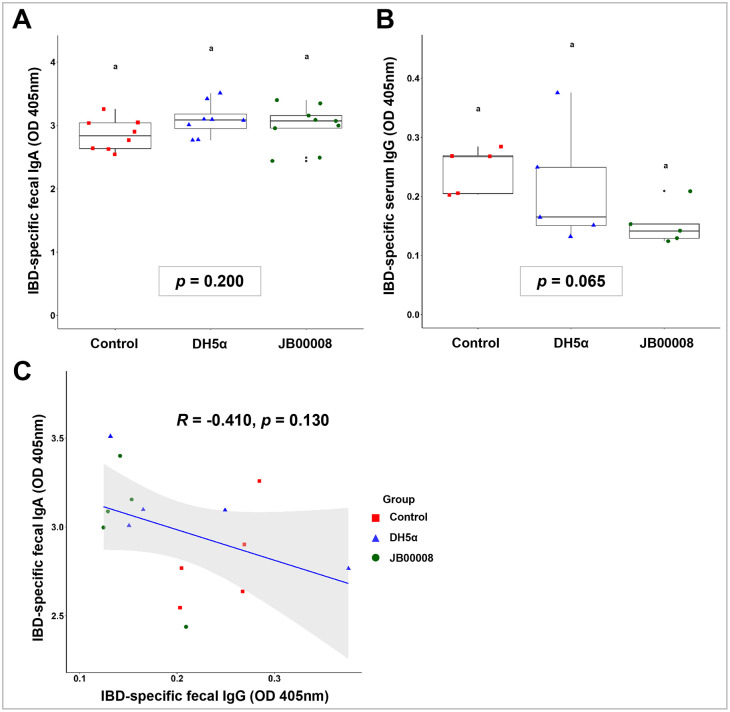
Efficiency of postbiotics on oral IBD vaccine response in chickens. (A) IBD-specific fecal IgA and (B) serum IgG. (C) Correlation between IBD-specific fecal IgA and serum IgG. Each point represents an individual bird sampled from the designated cage (Control, n = 8; DH5α, n = 8; JB00008, n = 9), and p-values were determined using the Kruskal–Wallis test followed by Dunn’s post hoc test. OD values were measured at 450 nm. The sample size (*n*) indicates the number of individual birds analyzed as observational units (subsamples) per treatment to assess within-unit physiological variance and immunological correlation.

At 13 days of age, IBD vaccination was administered. Two weeks post-immunization, anti-IBD fecal IgA and serum IgG levels were measured using ELISA to assess group-specific antibody responses. As shown in Fig 4A and 4B, there were no statistically significant differences in fecal IgA (*p* = 0.200) or serum IgG (*p* = 0.065) levels between the groups. Although the JB00008-fed group showed numerically higher IgA levels (OD450 = 3.02) than the PBS group (OD450 = 2.82), this difference was not statistically significant (p = 0.360). Additionally, a negative correlation was observed between fecal IgA and serum IgG levels, this correlation was not statistically significant (*p* = 0.130).

### Microbial diversity

**Fig 5:**
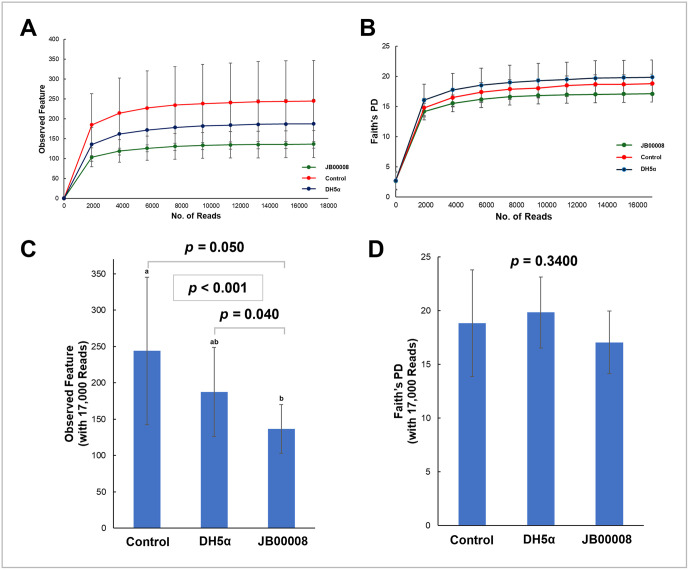
Alpha diversity of fecal samples from 28-day-old chickens. Observed features (A) and Faith’s PD (B) were calculated using different numbers of sequenced reads. Observed features (C) and Faith’s PD (D) were analyzed with normalization to 17,000 reads across 10 iterations. p-values were determined using the Kruskal–Wallis test. Bars represent the mean, and error bars indicate ± SD (Control, n = 10; DH5α, n = 10; JB00008, n = 9).

To assess the effects of pre-vaccination and post-biotic administration on gut microbial diversity and community structure, alpha diversity metrics, including observed features and Faith’s phylogenetic diversity (PD), were calculated. Rarefaction curves were generated using ten different read depths (Fig 5 (A, B)), and final analyses were conducted using 17,000 reads per sample. As the sequencing depth increased, the observed features gradually increased and plateaued beyond a certain threshold. At the final read depth, the observed features showed significant differences among all groups (*p* < 0.001). Although no significant difference was found between the control and DH5α groups, the JB00008 group showed significantly lower observed features than the control (136.67 ± 33.69 vs. 224 ± 101.43, *p* = 0.050) and DH5α (136.67 ± 33.69 vs. 187.6 ± 61.31, *p* = 0.040) groups. This suggests that the JB00008 group harbors significantly fewer distinct microbial taxa. Faith PD not differed among all groups (*p* = 0.340), and pairwise comparisons revealed no statistically significant differences.

**Fig 6:**
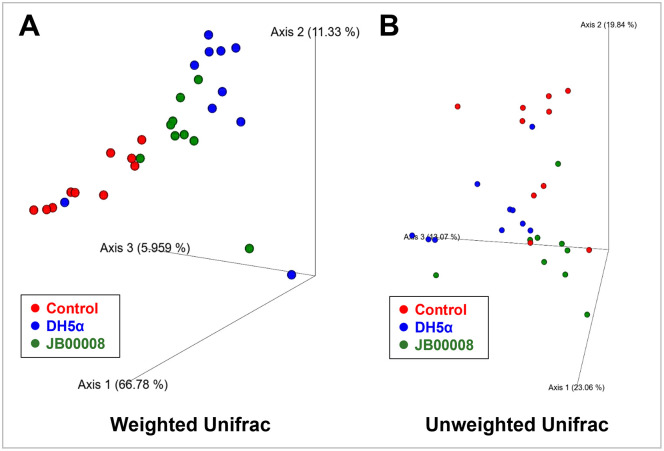
Beta diversity of fecal samples from 28-day-old chickens. Principal coordinate analysis (PCoA) plots were generated based on weighted (A) and unweighted (B) UniFrac distances. The percentages shown on each axis represent the variation explained by the respective coordinate. Each colored dot indicates an individual chicken sampled from the designated cage (Control, n = 10; DH5α, n = 10; JB00008, n = 9). The sample size (*n*) indicates the number of individual birds analyzed as observational units (subsamples) per treatment to capture within-unit micro-environmental and gut microbial community variation.

UniFrac distances were used to evaluate beta diversity and compare the gut microbial community composition among samples. These distances were visualized using principal coordinate analysis (PCoA) based on weighted (Fig 6A) and unweighted (Fig 6B) UniFrac metrics. Accordingly, two PCoA plots were generated. One plot was derived from the weighted UniFrac distances (Fig 6A), and the other from the unweighted UniFrac distances (Fig 6B). Both weighted and unweighted UniFrac analyses demonstrated a significant separation among the groups (PERMANOVA, *p* = 0.001). These findings indicate that group-level differentiation is driven by differences in microbial composition and species abundance, as well as the phylogenetic diversity of the gut microbiota.

### Relative abundance

The relative abundances of the gut microbial communities were compared to identify the microbial features that distinguished the three groups. As shown in Fig 8, 21 phyla were identified. At the phylum level, the combined abundance of Firmicutes, Actinobacteriota, Fusobacteriota, and Bacteroidota was 98.73%, 96.5%, and 97.57%, and the combined relative abundance of the 18 major genera was 76.76%, 88.02%, and 84.40% in the Control, DH5α, and JB00008 groups, respectively (Table 2).

**Table 2:** Relative abundance of major bacterial group.

	Control	DH5a	JB00008	*p*.value
Phyla				
Firmicutes	96.94 ± 2.53^a^	92.55 ± 2.56^b^	90.69 ± 2.72^b^	<0.001
Actinobacteriota	1.30 ± 1.54^a^	2.74 ± 1.36^c^	5.19 ± 1.89^b^	0.001
Fusobacteriota	0.04 ± 0.06^a^	0.04 ± 0.05^b^	0.82 ± 0.55^a^	<0.001
Bacteroidota	0.45 ± 0.41^a^	1.17 ± 0.41^ab^	0.87 ± 0.86^b^	0.008
Genera				
*Romboutsia*	29.36 ± 26.64	24.18 ± 7.29	34.02 ± 7.99	0.194
*Lactobacillus*	7.39 ± 3.06^a^	30.98 ± 13.62^b^	14.75 ± 6.53^b^	<0.001
*Staphylococcus*	0.83 ± 0.66^a^	3.03 ± 2.49^c^	10.49 ± 4.65^b^	<0.001
*Turicibacter*	3.14 ± 2.68	1.51 ± 0.62	2.54 ± 0.61	0.100
*Corynebacterium*	1.18 ± 1.43^a^	2.31 ± 1.19^b^	4.38 ± 1.75^a^	0.003
*Enterococcus*	0.88 ± 0.64^a^	0.87 ± 0.36^b^	3.17 ± 0.71^a^	<0.001
*Terrisporobacter*	0.32 ± 0.70^a^	0.33 ± 0.19^b^	2.30 ± 1.26^b^	<0.001
*Ruminococcus_torques_group*	2.71 ± 2.03^a^	0.58 ± 0.70^b^	0.22 ± 0.14^a^	0.003
*Clostridia_UCG-014*	4.77 ± 3.79^a^	1.23 ± 1.44^b^	0.54 ± 0.87^a^	0.008
*Escherichia-Shigella*	0.41 ± 0.53^a^	0.18 ± 0.09^b^	0.48 ± 0.19^a^	0.006
*Streptococcus*	0.09 ± 0.09^a^	0.22 ± 0.13^b^	0.91 ± 0.26^a^	<0.001
*Bacillus*	0.45 ± 0.54^a^	0.21 ± 0.26^b^	0.07 ± 0.11^a^	0.012
*Lactococcus*	0.14 ± 0.08	0.44 ± 0.88	0.06 ± 0.07	0.103
*Brachybacterium*	0.06 ± 0.07^a^	0.19 ± 0.06^c^	0.38 ± 0.15^b^	0.0001
*Brevibacterium*	0.03 ± 0.03^a^	0.12 ± 0.05^c^	0.24 ± 0.11^b^	<0.001
*Bacteroides*	0.20 ± 0.26^a^	0.17 ± 0.06^b^	0.05 ± 0.05^a^	0.008
*Faecalibacterium*	0.02 ± 0.03^a^	0.20 ± 0.22^ab^	0.06 ± 0.07^b^	0.028
*Lysinibacillus*	0.00 ± 0.01^a^	0.08 ± 0.06^a^	0.02 ± 0.07^b^	0.006

All data were obtained from all relevant reads. Values are presented as the mean ± standard deviation. ^a-c^Different superscript letters within the same row indicate statistically significant differences. P-values were calculated using the Kruskal–Wallis test and Dunn’s test were applied. The data and statistical inferences are based on individual birds analyzed as observational units (subsamples) from the designated cage (Control, n = 10; DH5α, n = 10; JB00008, n = 9) to capture individual variation in specific microbial communities.

**Fig 7:**
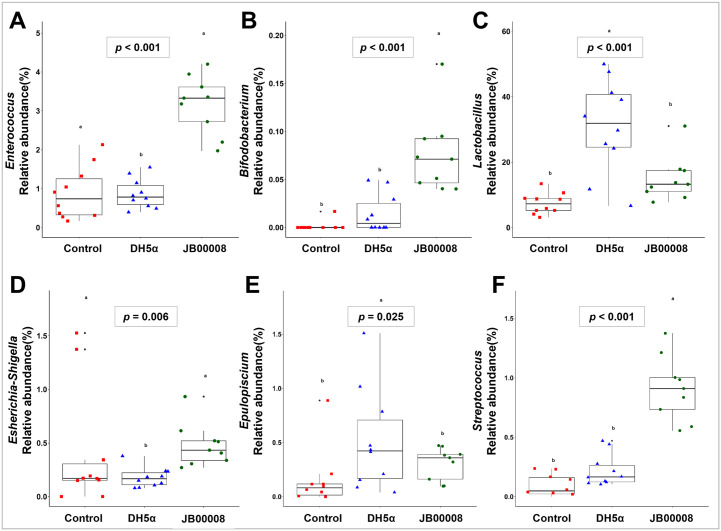
Relative abundance of significant genera. Six genera (A–F) were selected for visualization as boxplots. Each point represents an individual chicken sampled from the designated cage (Control, n = 10; DH5α, n = 10; JB00008, n = 9). P-values were calculated using the Kruskal-Wallis test followed by Dunn’s posthoc test. The sample size (*n*) indicates the number of individual birds analyzed as observational units (subsamples) per treatment to capture within-unit variation in specific gut microbial distributions.

Among these, two genera, *Lactobacillus* and *Romboutsia*, exhibited mean relative abundances exceeding 7.00% across all samples. The relative abundances of significantly different genera are shown in Fig 7. In the Control group, the *Ruminococcus* torques group (*p* = 0.003) and *Clostridia*_UCG-014 (*p* = 0.008) showed higher relative abundances than the other groups. In the DH5α group, *Lactobacillus* (*p* < 0.001) and *Lysinibacillus* (*p* = 0.006) were significantly more abundant than in the other groups. The genera *Staphylococcus* (*p* < 0.001), *Corynebacterium* (*p* = 0.003), *Streptococcus* (*p* < 0.001), *Brevibacterium* (*p* < 0.001), *Bifidobacterium* (*p* < 0.001), *Enterococcus* (*p* < 0.001), and *Epulopiscium* (*p* = 0.025) were significantly more abundant in JB00008 group, whereas *Escherichia-Shigella* showed no significant difference compared to the Control group (*p* = 0.055).

### Abundance of metagenomic feature

KEGG pathway analysis was performed to evaluate the functional differences in the gut microbiota according to the type of postbiotic supplementation (Fig 9). A total of 6,608 pathways were identified, and 11 KEGG pathways were identified by analyzing the differences using a linear discriminant analysis effect size (LEfSe; LDA score > 2.7 and *p* < 0.050). In the control group, pathways related to the putative ABC transport system ATP-binding protein and RNA polymerase sigma-54 factor showed high scores, and RNA polymerase sigma-32 factor in particular had a high LDA score of 3.0 or higher. In the JB00008 group, pathways related to iron complex transport showed higher scores than those in the other groups. Meanwhile, in the DH5α group, pathways related to the PTS system and 6-phospho-beta-glucosidase showed notably high scores.

**Fig 8:**
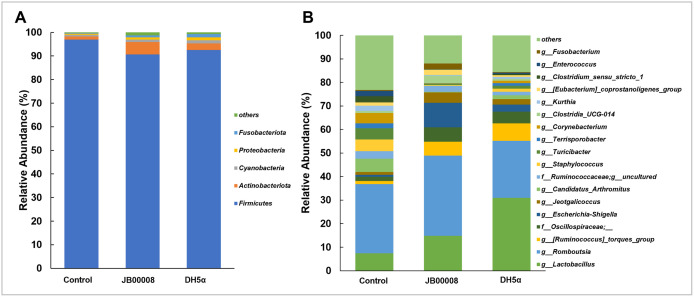
Relative abundance of major bacterial groups. Relative abundance is shown at the phylum (A) and genus (B) levels. Values represent the mean abundance of each microbial group.

**Fig 9:**
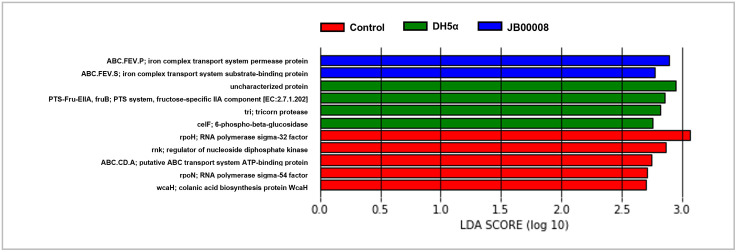
LEfSe analysis of KEGG Pathway. LEfSe analysis was performed using individual bird sampled from the designated cage(Control, n = 10; DH5α, n = 10; JB00008, n = 9). The sample size (*n*) indicates the number of individual birds analyzed as observational units (subsamples) per treatment to capture within-unit variation in the predicted functional profiles of the gut microbiota.

## Discussion

In this study, we evaluated the gut microbiome and immune response effects of postbiotics extracted from *E. faecium* JB00008 on physiological parameters, mucosal immunity, intestinal microbiota, and microbiota-derived metabolites in broiler chickens. Postbiotics did not significantly affect body weight but induced significant changes in immune gene expression and microbial diversity.

Some chickens exhibited a tendency to lose body weight after vaccination [21]. However, in this study, no significant differences in body weight were observed when the groups were fed different postbiotics prior to vaccination. This finding suggests that postbiotics play a role in preventing post-vaccination weight loss.

In this study, the group fed postbiotics showed significantly increased *IL-10* and *MUC2* gene expression. *IL-10* is an anti-inflammatory cytokine that plays an essential role in regulating inflammatory responses and maintaining the immune balance in the intestinal mucosa [11]. *IL-10* deficiency is closely associated with chronic inflammatory bowel disease (IBD). In this case, the group administered DH5α showed a significantly higher level of gene expression compared to the other two groups. This is thought to be mainly due to the effect of lipopolysaccharide (LPS) present in the outer membrane of *E. coli* DH5α activating Toll-like receptor 4 (TLR4) of immune cells, thereby inducing *IL-10* expression [22, 23]. In addition, the expression of *ANXA5* also significantly increased in the DH5α group, which seems to be because *ANXA5* binds to LPS and plays a role in activating M cells [24]. In other words, LPS provided by DH5α would have promoted the expression of *ANXA5* by activating M cells. Therefore, since both *IL-10* and *ANXA5* are activated by LPS, it is thought that the expression patterns of these two factors were similarly increased in the DH5α administration group. Furthermore, *MUC2*, a gel-forming mucin and a major component of the intestinal mucus layer, plays a key role in defending against pathogenic microbial invasion and physically protecting the intestinal epithelial cells [12]. The increased expression of *MUC2* in the JB00008 group is thought to be closely related to the activation of the intestinal mucosal immune system, thereby enhancing intestinal stability and absorption of vaccine antigens. Therefore, intestinal decrease in *IL-10* and *MUC2* levels correlates with an increased incidence of IBD, a chronic intestinal inflammatory disease [25, 26]. These results demonstrate that postbiotic supplementation can control intestinal inflammation and strengthen the mucus barrier, providing an important foundation for maintaining intestinal health and protecting against pathogenic infections in broilers.

IBD is an immunosuppressive and highly contagious disease caused by the IBD virus, which primarily affects young birds and leads to significant economic losses in the poultry industry. In this study, a 12-day cell extract was used as an adjuvant to enhance the immunological efficacy of IBD vaccination. Although IgG levels were lower in the other groups, these results suggest that increased IgA levels at specific sites can influence systemic IgG levels in some diseases. *ANXA5* is associated with M cell differentiation and efficacy of oral vaccines [17]. Studies have shown that increased *ANXA5* expression may be associated with enhanced M-cell function and immune responses to oral vaccines. However, in this study, despite higher *ANXA5* levels in the DH5α group, vaccine efficacy was not significantly enhanced, suggesting that increased *ANXA5* levels do not always contribute to vaccine efficacy. IL-6 is a key cytokine known to positively influence vaccine efficacy. It has been suggested that IL-6 contributes to oral immunity by acting as a mucosal immune adjuvant [27]. However, in this study, despite higher IL-6 levels in the DH5α and JB00008 groups, vaccine efficacy did not significantly increase. This finding suggests that excessive IL-6 elevation does not always contribute to increased vaccine efficacy.

In this study, the analysis of alpha diversity in the gut microbiome demonstrated that postbiotics administered prior to IBD vaccination influenced microbial diversity. No significant difference was observed between the Control and DH5α groups in observed features; however, JB00008 showed a significant difference compared to both groups, suggesting a distinct effect on diversity. Beta diversity analysis further revealed significant group separation based on the UniFrac distance, which was clearly visualized in both weighted and unweighted PCoA plots. These findings indicate that postbiotics are associated with changes in the gut microbial diversity and play an important role in distinguishing microbial community structures.

The increase in specific gut microbes observed in the JB00008 group provides important insights into enhancing immune responses and improving the intestinal health of chickens. Although *Streptococcus* is generally considered pathogenic to various avian and poultry species [28], certain strains have been reported to be positively associated with the immune function in chickens under specific environmental conditions [29]. Thus, administration of JB00008 may have stimulated the immune system by promoting the proliferation of these bacteria [30]. Recent studies have reported that *Staphylococcus* and *Enterococcus* species may exhibit symbiotic interactions that promote growth [31]. The differences in the relative abundances observed in this study and the results shown in S1Fig can be interpreted as a correlation between these abundances. *Escherichia-Shigella*, two representative pathogens, are known to invade intestinal epithelial cells, proliferate intracellularly, and induce diarrheal diseases [32]. Significant differences were observed between the three groups, with no significant difference between the control and JB00008 groups. Conversely, the DH5α group exhibited a relatively low abundance, showing a trend comparable to findings that certain non-pathogenic *E. coli* strains competitively suppress pathogenic bacteria. In this study, it can be assumed that DH5α reduced the abundance through competitive interactions with *Escherichia-Shigella*.[33]

Furthermore, Corynebacterium has been associated with immune regulation, including enhanced macrophage activity and the modulation of antibody responses [34], whereas *Enterococcus* has been reported to provide various physiological benefits to chickens, such as improved immune responses, suppression of *Salmonella* infection, weight gain, stimulation of Short-chain Fatty acids(SCFA) production, and inhibition of inflammatory cytokines [35]. Notably, the JB00008 group showed higher relative abundances of *Bifidobacterium* [36], *Epulopiscium* [37], and *Enterococcus*, which are key SCFA-producing genera (e.g., acetate and lactate). These findings suggest that JB00008 postbiotics contribute to intestinal homeostasis and immune modulation by enhancing microbial metabolism. Interestingly, supplementation with *Enterococcus*-derived postbiotics increased the relative abundance of *Enterococcus* in the gut, in contrast to previous reports showing decreased endogenous enterococci following the administration of live *Enterococcus* probiotics [10]. Collectively, these results indicated that JB00008 postbiotics may improve both intestinal health and vaccine-induced immune responses by beneficially modulating the gut microbiota.

We further investigated the functional changes in the gut microbiota following postbiotic administration using KEGG pathway analysis. In the Control group, increased pathways related to RNA polymerase sigma-54 and sigma-32 suggested enhanced regulatory mechanisms associated with stress responses and environmental adaptation, likely reflecting defensive responses to external stimuli or maintenance of physiological balance [38]. In the JB00008 group, the enrichment of iron complex transport pathways has potential roles in limiting the survival and colonization of pathogenic bacteria and strengthening host immune defenses [39], consistent with the antipathogenic properties of probiotics and postbiotics. Although no significant changes were observed in systemic IgA and IgG levels, these functional adaptations may contribute to maintaining mucosal immune balance and basal immune readiness [40, 41]. By contrast, the DH5α group exhibited increased activity in the PTS system and 6-phospho-beta-glucosidase pathways, implying enhanced carbohydrate uptake and degradation, which may contribute not only to energy acquisition but also to SCFA production [42]. These findings indicate that distinct postbiotic treatments modulate gut microbiota through different functional pathways, with JB00008 showing a potential association with immune-related microbial functions.

Lastly, we acknowledge the limited cage replication in this study, which may have introduced cage-associated confounding effects and restricted robust pen-level inference. Therefore, the observed individual-level physiological, immunological, and microbiological responses should be interpreted with appropriate caution as exploratory insights. Further studies incorporating a greater number of independent pens are warranted to validate these findings and improve their generalizability.

## Conclusions

This study suggests that *E. faecium* JB00008 postbiotics may contribute to the maintenance of intestinal barrier integrity and modulation of gut microbiota in vaccinated chickens. While the treatment did not significantly increase systemic antibody titers, it was associated with the upregulation of key mucosal integrity-related genes and alterations in microbial functional pathways, including pathways related to iron transport and microbial metabolism. These findings highlight the potential of JB00008 postbiotics as a functional feed additive to support gastrointestinal health and microbiota-associated homeostasis in poultry production. However, further studies involving direct viral challenge and long-term evaluation of protective immunity are required to fully elucidate the practical role of JB00008 in supporting host responses during vaccination.

## Supporting information

S1 Table Nutrient composition and ingredient formulation of the experimental diets. (TIF)

S1 Fig Pearson correlation between *Staphylococcus* and *Enterococcus.* A positive correlation between the relative abundance of Staphylococcus and Enterococcus was observed across samples. The correlation coefficient was calculated using Spearman’s rank test.
(TIF)
