# Supplementary figures and images for: Postbiotic effects of Enterococcus faecium JB00008 on gut health and IBD vaccination in broiler chickens
Source: PLoS One. 2026 Jul 16;21(7):e0353974. doi: 10.1371/journal.pone.0353974 (PMC13374999; doi:10.1371/journal.pone.0353974)

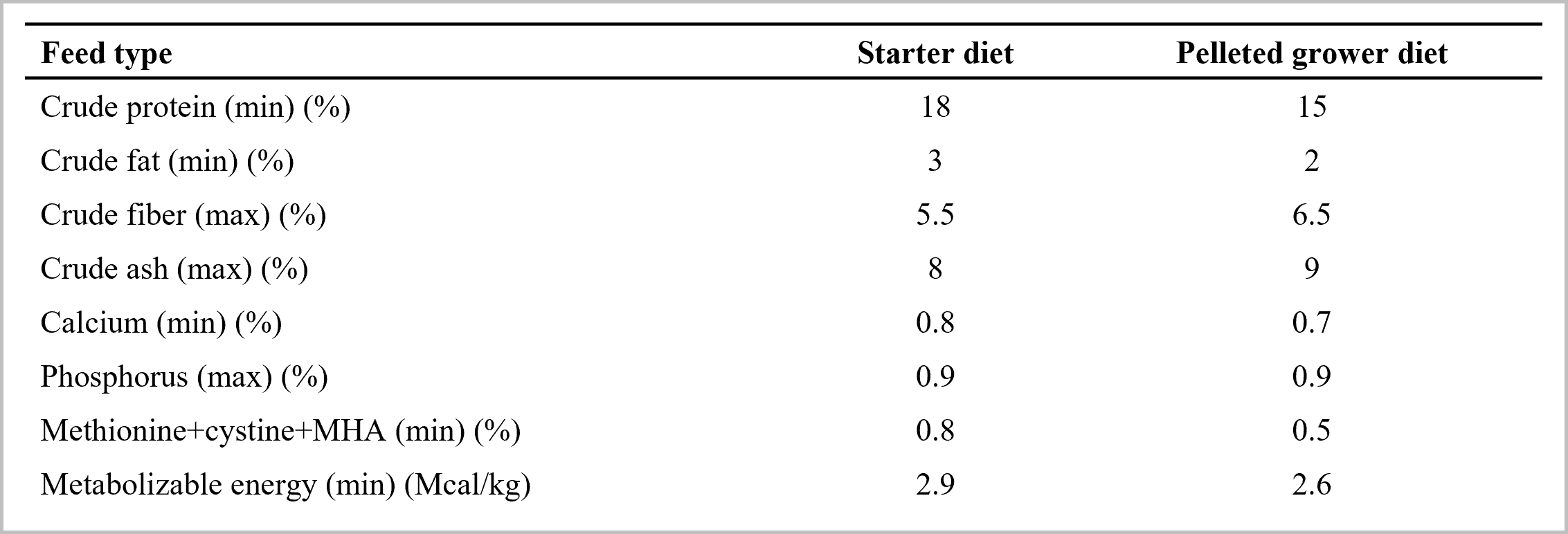

Supplement: S1 Table — (TIF) [file pone.0353974.s001.tif]

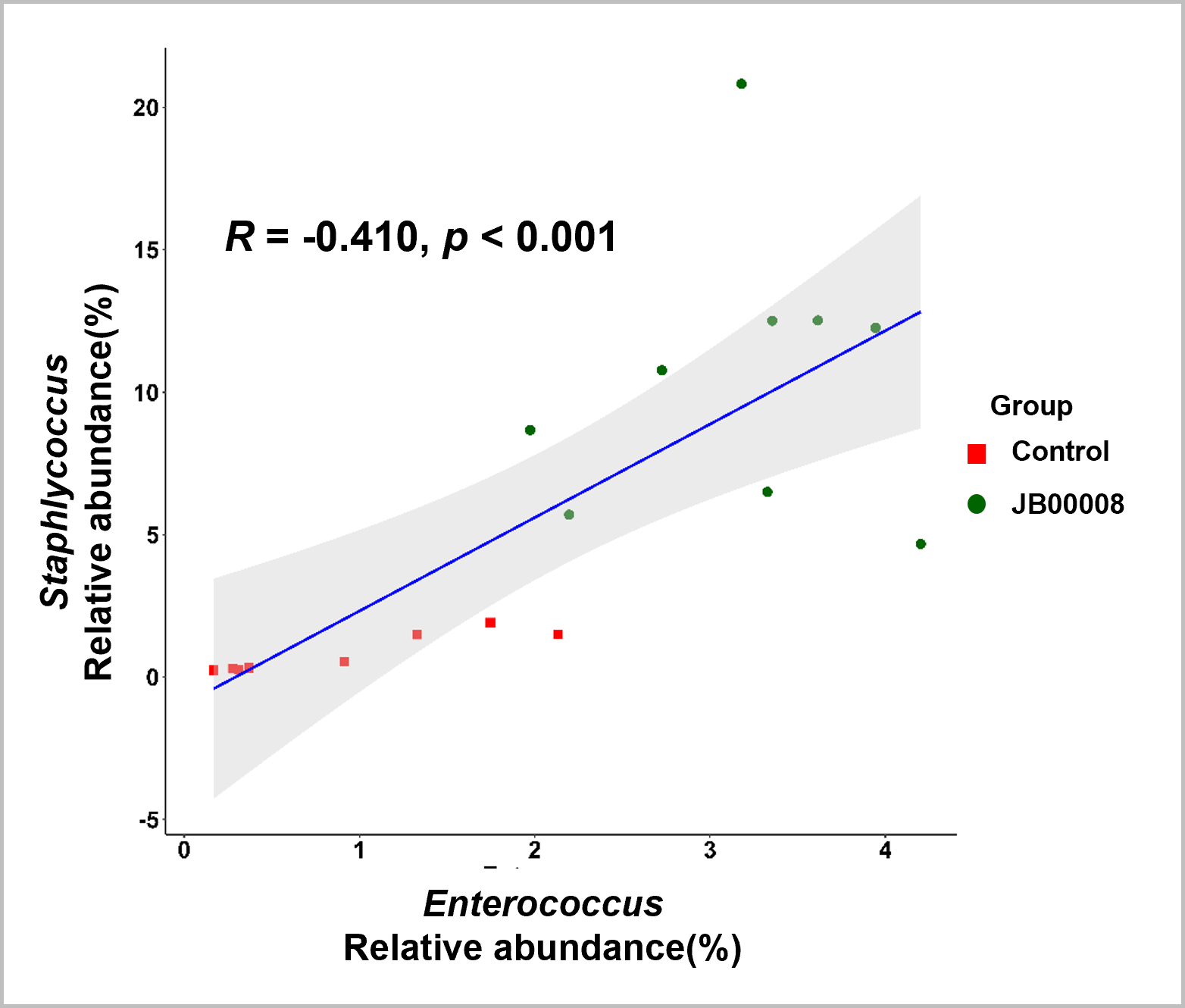

Supplement: S1 Fig — A positive correlation between the relative abundance of Staphylococcus and Enterococcus was observed across samples. The correlation coefficient was calculated using Spearman’s rank test. (TIF) [file pone.0353974.s002.tif]
